# Sensorimotor Oscillations in Human Infants during an Innate Rhythmic Movement

**DOI:** 10.3390/brainsci14040402

**Published:** 2024-04-20

**Authors:** Helene Vitali, Claudio Campus, Valentina De Giorgis, Sabrina Signorini, Federica Morelli, Marco Fasce, Monica Gori

**Affiliations:** 1 Unit for Visually Impaired People, Istituto Italiano di Tecnologia, 16152 Genoa, Italy; helene.vitali@iit.it (H.V.);; 2 Dipartimento di Informatica, Bioingegneria, Robotica e Ingegneria dei Sistemi (DIBRIS), University of Genova, 16145 Genoa, Italy; 3 Department of Child Neurology and Psychiatry, IRCCS Mondino Foundation, 27100 Pavia, Italy; valentina.degiorgis@mondino.it (V.D.G.);; 4 Department of Brain and Behavioural Sciences, University of Pavia, 27100 Pavia, Italy; 5 Developmental Neuro-Ophthalmology Unit, IRCCS Mondino Foundation, 27100 Pavia, Italyfederica.morelli02@universitadipavia.it (F.M.)

**Keywords:** sensorimotor, infants, non-nutritive sucking, brain oscillations, beta synchronisation

## Abstract

The relationship between cerebral rhythms and early sensorimotor development is not clear. In recent decades, evidence revealed a rhythmic modulation involving sensorimotor processing. A widely corroborated functional role of oscillatory activity is to coordinate the information flow across sensorimotor networks. Their activity is coordinated by event-related synchronisation and desynchronisation in different sensorimotor rhythms, which indicate parallel processes may be occurring in the neuronal network during movement. To date, the dynamics of these brain oscillations and early sensorimotor development are unexplored. Our study investigates the relationship between the cerebral rhythms using EEG and a typical rhythmic movement of infants, the non-nutritive sucking (NNS) behaviour. NNS is an endogenous behaviour that originates from the suck central pattern generator in the brainstem. We find, in 17 infants, that sucking frequency correlates with beta synchronisation within the sensorimotor area in two phases: one strongly anticipating (~3 s) and the other encompassing the start of the motion. These findings suggest that a beta synchronisation of the sensorimotor cortex may influence the sensorimotor dynamics of NNS activity. Our results reveal the importance of rapid brain oscillations in infants and the role of beta synchronisation and their possible role in the communication between cortical and deep generators.

## 1. Introduction

Natural behaviour relies on a dynamic interaction between multimodal sensorimotor loops coordinating the information flow and synchronising signals processed by different systems [1,2]. Sensorimotor activity is observed in the form of EEG event-related (de)synchronisation (ERD/ERS) [3]. The typical pattern of cortical activation in adults during a voluntary movement shows an ERD of mu (8–12 Hz) and beta (14–32 Hz) activity during the planning period (approximately between 3 and 1.5 s prior to the movement) and the motor execution, observation, or imagination (starting approximately 0.7 s before the movement) [4,5,6,7] over the regionally localised motor cortex specifically related to the part of the body that produces the movement [6]. The end of the movement is followed by a beta ERS called the beta rebound [8,9,10]. These findings originally led to the conclusion that desynchronisation is associated with an activation of sensorimotor areas, whereas synchronisation occurs when the cortex enter in an idling state. Instead, many later studies evidenced a deeper and more active role of beta synchronisation in the cortical-subcortical information processing [8,11,12,13,14].

The role of these rhythms in early life and how they differ from adults has not been fully explored. An adult-like neurophysiological pattern during active movement was found in older infants, suggesting the cortical networks learn to represent patterns of biological motion of human action early in life [15,16]. However, few studies have investigated sensorimotor neural activity in infants [15,16,17,18,19,20], leaving many mechanisms unexplored, such as the involvement of the beta synchronisation in infancy. Beta activity is physiologically underrepresented before the onset of maturation of the cerebral cortex and progressively emerges during the last trimester of pregnancy [21,22], thanks to the greater development of cerebral pathways and the organization of neuronal circuits [23]. Adequate quantity of beta activity is reflected in an optimal development of motor and attentional skills [18,24]. However, a better understanding of its role in the first years of life opens potential windows for early intervention. Therefore, here we want to investigate whether beta oscillations may be involved in cortico-subcortical communication already early in life.

An indirect way to measure it, following a neuroethological approach [25], is to explore the innate behaviours. Indeed, they are considered a window into the correct maturation of the central nervous system [26]. In this study, for the first time, we tried to understand what the communication mechanisms between brain structures could be by studying one of the first innate behaviours: non-nutritive sucking (NNS). Non-nutritive sucking is a spontaneous motor behaviour manifesting in a stable rhythm organised as a series of bursts of sucking with pauses in between [27]. The ontogenesis of NNS is primarily dependent on brain maturation and represents an excellent marker of optimal functioning of the central nervous system in the first weeks of life [28]. Specifically, it represents a compelling model of communication between cortical and deep generators of movement. NNS is generated by suck central pattern generators (sCPG) located in the brainstem reticular formation [26,29]. A CPG is operationally defined as a small network of neurons whose activity can generate specific movements with correct timing and sequences in the absence of sensory feedback [30]. While the pattern-generating circuit of rhythmic oral motor activity is located in the brainstem, the quality of sucking behaviour is modulated by cortical activity [27], while also integrating signals from external inputs [31,32,33,34].

As such, the NNS could be an interesting model to investigate whether beta activity plays a role in cortico-subcortical communication in the first years of life. Furthermore, the possible involvement of other frequency bands, such as the mu rhythm, might also play a role in this modulation. Specifically, we were wondering whether the mechanisms governing these innate movements were the same that regulate voluntary movements or if they are linked to spontaneous brain activity. In the first case, we would expect a desynchronisation response, while in the second case, a synchronisation. To address our objective, we simultaneously recorded a video and EEG in 17 typically developed infants and investigated the relationship between sensorimotor oscillations and NNS behaviour.

## 2. Materials and Methods

### 2.1. Participants

Our sample is composed of 17 subjects (5 females, median = 22 months, range = 6–42 months). None of the subjects had a history of prenatal infections, foetal distress during delivery, metabolic disorders, or CNS anoxic-ischemic-haemorrhagic injury. A total of two subjects in our sample were born preterm but none were extremely preterm (<32 weeks gestational age [GA]). All subjects had good general health status, and none had cognitive impairment based onclinical evaluation. Although the range of age could seem broad, NNS is stable and well-patterned by 36 weeks GA. Indeed, NNS starts in the early foetal period, between 15–18 weeks of GA [35] and develops during the gestational period; in the neonatal period, the temporal organization showes no qualitative changes. There is an increase in the sucking frequency (average rate per second per burst) from 1 to 6 months. Starting from 6 months, the babies continue to suck in the same non-nutritive mode [36]. Please see the Supplementary Materials for information about the raw data including the single subject age, NNS frequency and mu and beta activity (see Table S1), and see Figure S1, which illustrates the absence of correlation between age and NNS frequency.

### 2.2. Data Acquisition

All data were selected retrospectively from the clinical database of the epilepsy lab of Department of Child Neurology and Psychiatry, IRCCS Mondino Foundation. EEG data were acquired using a Nicolet vEEG 5.94 system with a 512 Hz sampling frequency. The montage had 21 scalp electrodes placed according to the International 10–20 System plus a physical reference placed in Fpz. Each session was EEG- and video-recorded with two infrared cameras. Video data were acquired with a sampling of 25 fps. For the entire session, the infant was made to sit (or lie down depending on age) on a bed positioned inside an isolated EEG recording room under dark and silent conditions. The bed has an ergonomic seat that reduces the possibility of movement from the original position, both to reduce motor artifacts and for safety reasons. An operator continuously monitored the state of the participant. Ethical review and approval were not required in accordance with the local legislation and institutional requirements. However, a medical supervision was conducted and a written informed consent to record video-EEG and to be part of this retrospective research was signed by the participants’ legal guardians/next of kin.

In a later scoring phase, for global power spectral analysis, we selected 3 min, mean duration = 183 s, 95% CI = [175, 191] s, of EEG trace during the resting-state period (without NNS) for each subject, which we have considered as a baseline. We utilized the best conditions to record resting state in infants [37], using sensory deprivation conditions (e.g., in the dark and in silence) during which infants had their eyes open, and by excluding periods from the analysis in which children were moving or falling asleep. Moreover, we selected video-EEG of five bursts (when possible) during NNS activity (with pacifier or thumb), with a mean duration for each participant = 31 s, 95% CI = [23, 39] s. Specifically, the sucking condition included EEG recording, during which infants produce a visible NNS behaviour.

For a single-trial analysis, we selected the sucking bursts in 30 min of the EEG trace for each infant. For each sucking burst, we determined the number of sucks per burst (SPB) from the video synchronized with the EEG trace. The number of SPB were counted from the video in slow-motion mode by two operators independently naive to the purpose of the study. The raters agreed on trials where there were divergent ratings for the sucks per burst. Then we calculated the sucking frequency (Hz) as the number of SPB on the burst time (in seconds). To avoid the possibility that other types of movements will affect mu and beta activity, we excluded trials in which it was evident form the video that infant was making excessive movements with a different part of the body (e.g., leg movements, grasping, etc.). We also excluded all trials that had an intertrial time of less than 4 s. The final mean number of burst trials = 7.53, CI = [2, 17]; the mean duration(s) of burst trials = 6.67, CI = [2.83, 14.58]; and the mean SPB = 14.58, CI = [5.67, 31.12].

### 2.3. Pre-Processing

We applied both a notch and a band-pass filter between 0.16 and 100 Hz, then a subsampling to 256 Hz to the EEG data. The EEG signal was re-referenced to the average. During the pre-processing phase, transient high-amplitude artefacts were removed using artefact subspace reconstruction (ASR), which is an automated artefact rejection method available as a plug-in for EEGLAB software [38,39]. ASR uses a sliding window technique whereby each window of EEG data is decomposed via principal component analysis and compared with data from a clean baseline EEG recording. The ASR algorithm identifies principal subspaces that deviate from the baseline; it then reconstructs these subspaces using a mixing matrix computed from the baseline EEG recording. In our study, we used a sliding window of 500 ms and a threshold of three standard deviations to identify corrupted subspaces. In addition, the channels that remained heavily artifacted were removed following a visual inspection. In two EEG traces, we also applied ICA (independent component analysis) to remove artefacts related to ocular activity and problems with electrodes. Missing or electrodes containing artefacts were interpolated.

### 2.4. Region of Interest (ROI) and Frequency Selection

For each analysis, we considered the spectral activity of the C3 and C4 channels, which better represent the region of our interest (ROI) in the sensorimotor area. We specifically investigated the spectral activity for mu1 (8–10 Hz), mu2 (10–12 Hz), and beta (14–20) bands, which are the typical bands analysed in studies that investigated motor movements. However, previous studies showed that in older infants and children’s action execution, observation with and without a social context results in EEG power suppression in the 6–9 Hz range [15,40,41,42]. These studies define this frequency range as shifted mu activity in infants representing an EEG signature of the ‘mirror-neuron’ systems. The definition of a low-frequency mu activity comes from studies on the background activity maturation, which shifts towards higher frequencies throughout childhood, stabilizing at the typical alpha bands during adolescence [43]. A recent study revealed that two different spectral peaks in the first three years of life exist in the occipital area, one on a slow frequency band that shifts through fast frequencies during the development and a second peak on the typical alpha1 band [44]. The mu rhythm also found a similar pattern, although the prominent mu peak corresponds to the higher alpha band [45]. The association between the mu peak and the higher alpha band was found in children and adults, showing that in the first three years of life, the mu activity has already reached a clear 8–9 Hz peak [45]. Based on previous statements, we maintained the typical adult’s spectral band to evaluate mu activity. However, investigating all the EEG bands, the slower frequencies were considered. Moreover, studies on adults have found a role of beta rhythm that is poorly considered in infants. The few studies that investigated the beta activity in infants revealed that typical adult beta activity also responds to action processing in early life [15,16]. These studies evidenced that beta peak in infants is around 18 Hz; therefore, we considered only the first adult’s beta sub-bands (14–20 Hz). Further ROIs and frequency bands are sometime considered as controls (see Section 2.6).

### 2.5. Statistics

#### 2.5.1. Global Spectral Power Analysis

From each subject’s cleaned data, we computed power spectral density (PSD) expressed in (μV^2^/Hz). To this end, we applied the *spectopo* function of EEGLAB, which returns a PSD estimate via Welch’s method. The signal was divided into sections with a duration of 1 s with an overlap of 50%. Each section was windowed with a Hamming window, and modified periodograms were computed and averaged. Then, we considered spectral activity for mu1 (8–10 Hz), mu2 (10–12 Hz), and beta (14–20) spectral bands. To make spectral activity comparable among subjects, we normalized it by computing the relative spectral power (expressed in %) that measures the ratio of the total power in the band (i.e., absolute spectral power) to the total power in the signal. In both analyses, we considered all spectral bands in the sensorimotor area, calculated by averaging the spectral power of the C3 and C4 channels. We performed an independent linear regression for each selected spectral band, considering sucking frequency as a dependent variable and relative band power as an independent variable. Specifically, in the first analysis, we explored a correlation between baseline activity in the selected spectral band frequencies and sucking frequency. In the second analysis, we checked the possible relationship between brain activity during sucking and sucking frequency to see how the motor activity was modulated. We considered the results significant only when *p* < 0.05 and R^2^ > 0.30.

#### 2.5.2. Single Trial ERSP Analysis

From each subject’s cleaned data, we computed event-related spectral perturbation (ERSP). We segmented data from 4 s before to 2.5 s after the sucking onset. For each segment, the period from 3 to 4 s before the onset was used as a baseline. We calculated ERSP using the *newtimef* function of EEGLAB and applying the full-epoch length single-trial baseline correction methods; these methods normalise the spectral activity of each epoch, first using the mean spectral activity of the epoch and then the mean activity of the baseline period. The advantages of this approach for this particular study are related to the fact of carrying out an analysis on single trials, an approach that is not typically used in ERSP studies. This method minimises the contribution of artefactual data trials with high-amplitude spectral estimates and is robust to outliers when performing statistical inference testing [46].

We specifically extracted ERSP from 6 to 32 Hz using a Morlet Wavelet, starting with three cycles and increasing with a 0.1 factor. We computed mean ERSP of considered bands on the sensorimotor area and within 0.5 s time-windows from 3 s before the start of sucking to 2.5 s after with respect to the sucking onset.

We fitted a series of linear mixed models (LMMs) independently for the ERSP of each band. The model fitting was undertaken using the *lmer* function of the lme4 package [47]. The LMMs predictors were evaluated using Type III Wald χ^2^ tests as implemented in the *Anova* function of the car package [48]. We included random intercepts considering within-subject coefficients in all models, allowing for individual differences, but did not have enough power to estimate participant-specific slopes. We considered sucking frequency (SF) as the dependent variable. We evaluated the effects and interactions of Time Window, ERSP, and Age (in years). We did not find any significant interactions with Age, so to increase statistical power we removed it from the model and the following analyses. Significant fixed effects were further investigated with the *testInteractions* function of the phia package [49] by obtaining estimated marginal means (EMMs) and slopes by computing their contrasts. Effects were retained as significant when *p* < 0.05 after Bonferroni correction. The effect size (partial eta square, η_p_^2^) of each linear mixed model effect was calculated using the *F_to_eta2* function and effect size package [50]. According to Wilkinson’s notation [51], adopted by lme4 package [47], the models fitted were:

SF ~ Time Window ∗ ERSP ∗ Age + (1|Subject)(1)

SF ~ Time Window ∗ ERSP + (1|Subject)(2)

where the tilde is the operator (~) that separates the left side of an equation in which dependent variables are presented (e.g., sucking frequency) from the right side in which the fixed effects are considered (e.g., Time Window and ERSP, and Age). The piece of the formula (1|Subject) represents the inclusion of random intercepts considering within-subject coefficients in all models.

Finally, to exclude the increased beta activity due to muscular and swallowing artifacts in the EEG signals, we performed further analysis on temporal channels (T3, T4), which are more affected by these kinds of artifact. Generally, in infants, the muscle artifacts from jaw and arm movements appear mainly in peripheral electrodes [52]. Therefore, to exclude that our results are an effect of artifact movement we also considered a control ROI that included more peripheral electrodes. We did not find any significant interaction between time-window and mu1 (F(10) = 1.1, *p* = 0.39, η_p_^2^ = 0.009), mu2 (F(10) = 0.8, *p* = 0.61, η_p_^2^ = 0.007), and beta(F(10) = 0.6, *p* = 0.81, η_p_^2^ = 0.005) ERSP, suggesting that our results specifically involved the central area.

### 2.6. Control Analyses

To control for the potential of infant movements artifacts in the data selection, we added a control ROI to further exclude an effect of muscular artifacts. In addition, we adopted a normalisation that minimises the contribution of artefactual data trials in the ERSP analysis.

We also evaluated an effect of age. As specified in the *participants* section, the NNS is fully matured at six months of age and does not seem to change with development. This agrees with our data, as shown in Figure S1. However, many changes occur in the maturation of spectral background activity in the first years of life. Although we maintained the typical EEG band for the reasons described in the Section 2.4, we added further analysis to investigate the slow mu activity in the 6–9 Hz band and the spectral peak frequency of each single subject. The global spectral power analysis showed no significant correlation between relative power in 6–9 Hz band and sucking frequency both during baseline, (R^2^ = 0.003) and sucking period (R^2^ = 0.009). Moreover, we considered the peak frequency of background activity to investigate possible association between sucking frequency and EEG maturation. To reliably estimate it, we adopted a three-stage approach. In the first, we searched for the peak in the raw spectrum. In the second, we searched for the peak in the spectrum after it was detrended using an exponential decay function. For this purpose, we considered the logarithm of the PSD at different frequencies and fitted a linear regression model: log(PSD) = A + B·Frequency. Therefore, we computed the PSD using coefficients A and B estimated by the regression, PSDest = exp(A)·exp(B·Frequency), and we searched for the peak in the detrended PSD PSDdetrend = PSD − PSDest. We used the findpeaks function of the pracma [53] package for R [54] to identify peaks in the background in the raw and the detrended spectra. In the third approach, we compared automatic peak estimates from stages one and two and where they differed (most times, they matched). Finally, we selected the most reliable peak position through visual inspection in case the automatic peak estimates from stages 1 and 2 differed. However, data did not show any significant correlation between sucking frequency and peak frequency, with all the three measures, respectively: R^2^ = 0.2, R^2^ = 0.1, R^2^ = 0.1.

### 2.7. Data Availability

The datasets generated during this study are available on Zenodo: https://zenodo.org/records/10700951 (accessed on 24 February 2024). The processing and analysis pipeline performed publicly available function of tools (EEGLAB: https://sccn.ucsd.edu/eeglab/index.php (accessed on 24 February 2024), R: https://www.r-project.org (accessed on 24 February 2024)).

## 3. Results

**Figure 1:**
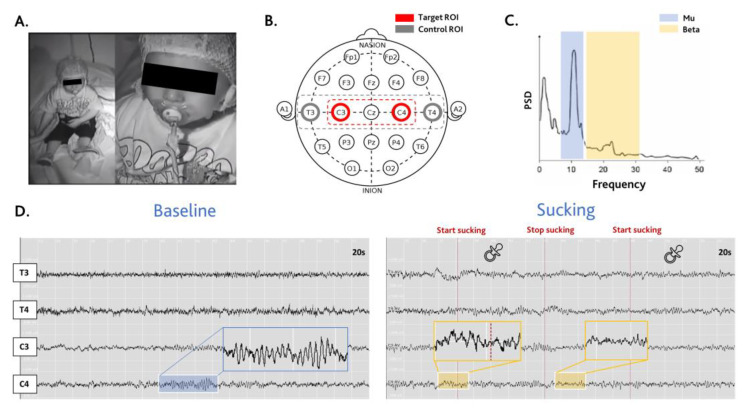
Methods. (**A**) View at the infra-red camera during the recording. (**B**) Illustration of International 10–20 System of EEG electrodes. The red and grey cycles represent the selected electrodes (C3, C4, T3 and T4). The area selected by the red dashed line represent the target region of interest (ROI) analysed. The area selected by the grey dashed line represent the control region of interest (ROI) analysed. (**C**) Illustration of typical EEG power spectral density (PSD) plot. Blue and yellow areas highlight the EEG rhythms, respectively mu (8–12 Hz) and beta (14–32 Hz). (**D**) Representative EEG traces of baseline and sucking conditions. ((**D**), left) Baseline condition is characterized by an EEG recording of a resting-state period sensory deprivation environment during which infants had eyes open. ((**D**), right) Sucking condition is characterized by an EEG recording during which infants produce a visible NNS behaviour (with pacifier or thumb). Blue and yellow areas highlight examples of mu and beta the EEG rhythms, respectively.

Seventeen healthy infants participated in a video-EEG recording including a period of resting state and a period of natural NNS behaviour. We estimated the frequency of each sucking burst from the video synchronized with the EEG. We analysed EEG oscillations, as shown in Figure 1. Central derivation records the activity of the sensorimotor cortex. Sensorimotor activity is observed in the form of ERD/ERS of mu and beta brain rhythms. Therefore, we selected specific that bands for our global spectral power and single-trials ERSP analyses.

### 3.1. Global Spectral Power Analysis

**Figure 2:**
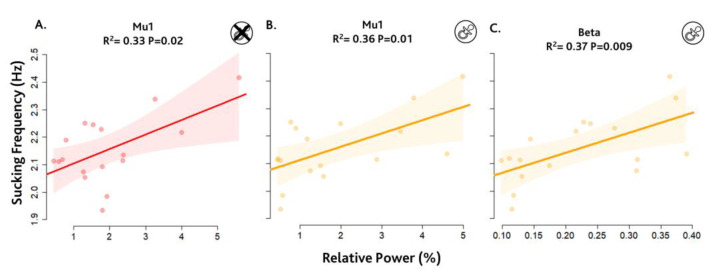
Global power spectral analysis. Figures show the significant correlations between relative spectral power (expressed in %) in mu and beta of the EEG sub-bands during baseline or during sucking conditions and sucking frequency. In red is represented the significant results (*p* < 0.05 e R^2^ > 0.30) related to baseline condition and in orange the results related to sucking condition. Each point represents a single subject. The shaded areas represent the 95% CI. (**A**) Correlation between sucking frequency and relative power of mu1 activity (8–10) Hz during resting-state. (**B**) Correlation between sucking frequency and relative power of mu1 activity (8–10) Hz during sucking condition. (**C**) Correlation between sucking frequency and relative power of beta activity (14–20) Hz during sucking condition.

First, we investigated the cortical rhythms from a global point of view and a possible relationship with sucking frequency (SF). Specifically, we correlated SF with resting-state brain activity (baseline) and brain activity during the sucking period. We found a positive correlation between SF and baseline brain activity for mu1 (8–10 Hz) rhythm. Thus, the power of mu1 oscillation is higher during baseline in children who exhibit higher mean sucking frequency. The result of the linear regression analysis is represented in Figure 2A. It reports the correlation between SF and mu1 relative power with a significant association (R^2^ = 0.33, *p* = 0.02). No significant correlation was found for mu2 (R^2^ = 0.001) and beta (R^2^ = 0.002).

Then, we explored how brain oscillations are involved in the modulation of motor activity, correlating SF with brain activity during the sucking period. Figure 2B,C show a positive correlation between SF and the relative power of mu1 and beta rhythms. Figure 2B reports the significant association related to mu1 (R^2^ = 0.36, *p* = 0.01). This result is in line with the baseline association, supporting that the power of mu1 oscillation is higher in children with higher mean sucking frequency and also during sucking behaviour. Figure 2C shows the significant association between sucking frequency and relative power related to beta (R^2^ = 0.37, *p* = 0.009). No significant correlation was found for Mu2, R^2^ = 0.06.

### 3.2. Single Trial ERSP Analysis

To investigate the brain dynamics associated with the NNS behaviour and to better understand the idling-synchronisation mechanisms, which we found in the global analysis, we explored the time-frequency properties at a single-trial level. We selected the same rhythms analysed above: mu1 (8–10 Hz), mu2 (10–12 Hz), and beta (14–20 Hz). For the temporal investigation, we considered 0.5 s time windows from 3 s before the start of sucking to 2.5 s after with respect to the sucking onset. For each band, we separately performed a linear mixed effect model analysis, considering the sucking frequency (SF) as a dependent variable, the band ERSP [46] as independent variables, and the subject as a random effect.

**Figure 3:**
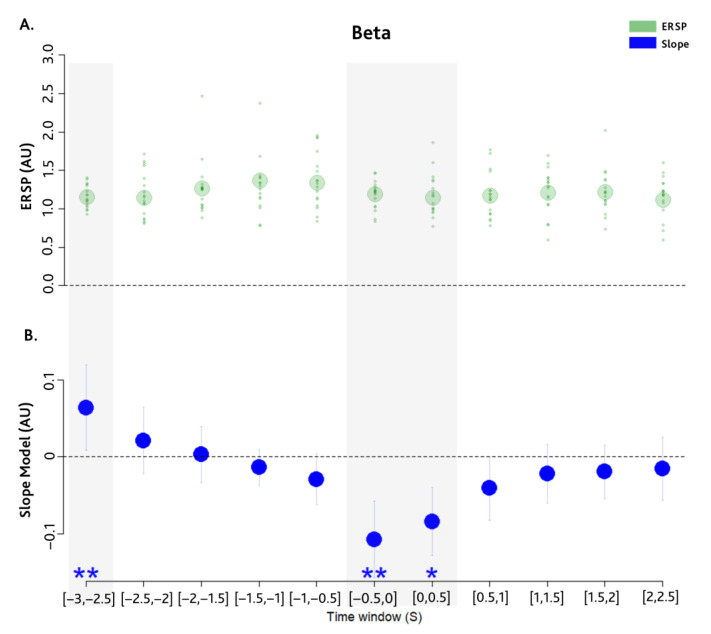
Temporal pattern of the beta ERSP and sucking frequency. This pattern emerges from single trial ERSP analysis adopting the normalization described in [46] and expressing ERSP in arbitrary units (AU). 0.5 s time-windows were considered from 3 s to 2.5 s after with respect to the sucking onset. The shaded grey areas represent the significant time-windows emerging from the model. (**A**) Plot of mean ERSP (larger transparent green circles) and single subjects ERSP (smaller green dots) in each analysed time window. The dashed line points out y = 0 highlighting that all points are positive. (**B**) Plot of the slopes estimated by the linear mixed model (blue points) and related 95% CI (vertical line) in each analysed time windows. The dashed line points out y = 0 highlighting positive and negative correlations. * indicates *p* < 0.05, ** indicates *p* < 0.01.

We found a significant interaction between beta ERSP and time-window, F(10) = 3.67, *p* < 0.0001, η_p_^2^ = 0.04. Post hoc tests showed the association between SF and ERSP in different time windows and showed (see Figure 3B) a significant positive correlation in the earliest [−3, −2.5] s time window, χ^2^ (1) = 11.07, *p* = 0.009; as well as a negative correlation encompassing the sucking onset, i.e., in the [−0.5, 0] s and [0, 0.5] s time-windows, respectively χ^2^ (1) = 13.34, *p* = 0.003 and χ^2^(1) = 8.68, *p* = 0.03. Thus, two different periods emerge as crucial for sucking modulation: an early period that starts 3 s prior to the movement and a late one, which starts just before the sucking and continues just after it (−0.5, 0.5 s). However, as shown in Figure 3A, ERSP is always positive, revealing that the sucking behaviour is modulated by a beta synchronisation. Specifically, an increase in beta synchronisation in the early time window and a reduction of beta synchronisation in the late period are associated with an increase in sucking frequency.

No significant interaction between ERSP and time-window was found in mu1, F(10) = 0.81, *p* = 0.6, η_p_^2^ = 0.007, and mu2 F(10) = 0.95, *p* = 0.5, η_p_^2^ = 0.008, frequency bands.

## 4. Discussion

The current study investigates the relationship between cortical oscillations and non-nutritive sucking (NNS) behaviour. We find that sucking activity is governed by synchronisations, suggesting that it is regulated by innate spontaneous activity rather than by the same mechanisms that regulate voluntary movements. Specifically, sucking frequency relates with beta synchronisation within the sensorimotor area in two different phases, one an early period, which starts 3 s prior to the movement, and a late one, which starts just before the sucking onset and continues just after it (−0.5, 0.5 s, respectively). An increase in beta synchronisation in the early time window and a reduction of beta synchronisation in the late period are associated with an increase in sucking frequency, suggesting a specific beta cortical-subcortical communication in NNS behaviour. Moreover, we find a general significant correlation between mu1 rhythm both during baseline and sucking period. If our findings were only due to stable individual differences in power spectral composition of the EEG signal, we would have expected to find only a generic association between spectral power in baseline and sucking frequency, which is not the case. Therefore, we speculate a potential specialized role of beta frequency band, which could come into play in regulating internal rhythms with external cues.

### 4.1. Beta Communication Model during NNS

There is evidence of beta oscillations involvement in a functional loop that connect the motor cortex with subcortical structures (e.g., basal ganglia, cerebellum, subthalamic nuclei, and peripheral motor units), supporting a beta cortical-subcortical information processing [11,14,55,56]. Similarly, our results suggest that a similar cortical-subcortical information processing could also occur correlate with to non-nutritive sucking behaviour. Indeed, NNS is generated by suck central pattern generators (sCPG) located in the brainstem reticular formation [29], while the quality and stability of sucking behaviour are modulated by more cranial structures (e.g., cortico-spinal) [27].

Specifically, the involvement of beta synchronisation in the patterns of biological motion of human action is associated with the gating probability [11] and sensorimotor dynamics (integrative information processing) [8,12]. To synchronise the sensory and motor signals, the beta oscillations seem to promote the actual motor set [57], enabling an efficient feedback processing, which is necessary to monitor and recalibrate the sensorimotor system [8,14,56,58]. We interpret the characteristic double-stage temporal communication of the sCPG-cortical functional loop during NNS (as shown by our results) as support for the role of beta oscillations in regulating sensorimotor activity. In detail, we find two phases that are involved in sucking regulation: an early one, in which ERSP positively correlates with sucking frequency, and a late one (around the beginning of the sucking period), in which ERSP is negatively correlates with sucking frequency. We propose that the increase of beta synchronisation in the early time window represents motor gating, which signals the alteration of the existing motor set. Moreover, the reduction of beta synchronisation just before the sucking, and that continues just after, is related to monitoring and recalibrating processes of the sensorimotor system.

During sucking behaviour, this beta synchronisation comes into play at the beginning of the movement, maybe because the first motor generator is not the motor cortex but is the sCPG. The presence of a deep generator (i.e., sCPG) that initiates the motor behaviour itself makes the motor cortex a second player. Specifically, the motor generators modulate the gating probability in an early phase, and then a recalibration of the sensorimotor dynamics. We can speculate that the gating probability is controlled by bottom-up communication increasing beta synchronisation in the sensorimotor cortex. The increased beta synchronisation identifies an alteration of the existing motor set and fosters, in the late time window, the ongoing neural mechanisms that allow the infant to prepare and adapt for the new motor set. This synchronisation is triggered by spontaneous sCPG activity, which then promotes NNS. In fact, the CPG sends the start of the movement signal to the periphery, and the information is also communicated to a higher level (i.e., sensorimotor cortex). The sensorimotor cortex then acts as a higher-level check-point system that coordinates and integrates different sensorimotor networks. Indeed, as we can see in the mixed model analysis 500 ms after the beginning of the sucking movement, cortical oscillations do not show a specific temporal role. The sucking behaviour is self-sustaining, underlying the cortical role only in the monitoring, and eventually recalibrating, phase.

### 4.2. Mu Oscillations

Our results only show a correlation between mu1 (8–10 Hz) during baseline and sucking frequency. This suggests that this rhythm does not have a strong role in the sensorimotor dynamics of natural sucking activity. Nevertheless, the positive correlation with baseline and sucking mu oscillations may suggest that this rhythm could be called into play in regulating the internal rhythms with external cues. Indeed, different studies showed that sensory stimulation can alter suck patterning in infants [31,32,33,34]. Second, the ongoing brain dynamics can be synchronised or phase reset by exogenous cues [59]. Third, the 8–10 Hz frequency band is generally more related to sensory-perceptual systems, typically called alpha rhythm for the visual system, mu rhythm for the somatosensory system, and tau rhythm for the acoustic system [60,61]. Fourth, it has already been suggested that mu activity intervenes in the modulation of the movement-related sensory feedback [7].

### 4.3. The Limitations and Strengths of the Study

The study is novel in the approach to studying neural mechanisms (i.e., following the neuroethological approach) and in the methods (i.e., single-trials time-frequency analysis) used. However, there are some limitations that should be addressed.

The single-trials time-frequency analysis is a better approach to capture transient burst activities than averaged-trials analyses, which can obscure such activity. Indeed, it is possible that the beta communication during sucking behaviour is not regulated in terms of sustained oscillatory brain activity, but originates from transient spontaneous bursts activity [62]. These bursts of high-power oscillations seem to represent the activation of internally generated events and facilitate the coordination between internal and external stimuli [63]. Additionally, recent research has indicated that infant sensorimotor activity is better described by burst-type beta oscillations [18,19]. Therefore, although our results on single trials still offer insight into the temporal changes crucial for understanding the role of beta activity in motor processing, further analysis employing more specific methods for single burst analysis is warranted [19].

Another potential limitation to the current paper is the sample size, which was not high for a correlative analysis; however, it is important to consider that even if we consider typical subjects, it is particularly difficult, while applying advanced cleaning techniques, to extract periods characterised by both clear sucking behaviour and clean EEG data. However, the size of the explored effects resulted sufficiently strong to be detected with the considered sample size. Nevertheless, we can exclude any possible spurious effect from muscle activity related sucking behaviour for different reasons. First, we preliminarily excluded events with muscle artifacts. Second, in the ERSP analysis, we used a normalisation that minimises the contribution of artefactual data trials. Third, we added a control ROI to further exclude an effect of muscular artifacts.

## 5. Conclusions

In conclusion, our study used a new approach to investigates the neural dynamics to understand the role of beta corico-subcortical communication processing. Specifically, we explored the relationship between cortical oscillations and the non-nutritive sucking behaviour, revealing a specific temporal dynamic of beta modulation. We speculate there is a unique coordination of the sCPG-cortical functional loop in the regulation of sucking behaviour. This would suggest that the complexity coordination in sensorimotor networks is already developed in infants. Further investigation into brain dynamics and the intricate role of mu and beta oscillations during complex sensorimotor behaviours are needed. These studies should delve into how sensorimotor oscillations are finely tuned over time as infants engage in voluntary motor tasks during NNS, or as external cues influence NNS. This line of inquiry promises to unveil the intricate interplay between resting-state networks and the external world.

## Data Availability

The datasets generated during this study are available on Zenodo at the following link: https://zenodo.org/records/10700951 (accessed on 24 February 2024). The processing and analysis pipeline were performing functions of tools that are public available (EEGLAB: https://sccn.ucsd.edu/eeglab/index.php (accessed on 24 February 2024), R: https://www.r-project.org (accessed on 24 February 2024)).

## Supplementary Materials

The following supporting information can be downloaded at: https://www.mdpi.com/article/10.3390/brainsci14040402/s1. Table S1. Individual subject data. Individual subject data at different ages showing the specific non-nutritive sucking (NNS) frequency and relative power of mu and beta activity during baseline and sucking period. Figure S1. Correlation of sucking frequency with age. No significant correlation was found between sucking frequency (Hz) and the subjects age (y), R^2^ = 0.05, *p* = 0.4. Points represent individual subjects, while continuous line represents the best linear fit. Figure S2. Time-frequency plot. x-axes represent time (S) and y-axes frequency (Hz). The red lines delimit the time-frequency bins resulted significant from the linear mixed model analyses. (A) Example of heat map obtained from typical subject (C008, age = 1.9). (B) Heat map that includes all subjects. Figure S3. Correlations for each time window. Significant associations were found between relative beta ERSP (arbitrary units, AU) and sucking frequency (Hz). Each point represents a single trial, while continuous lines represent the best linear fit. (A–C) show the associations in the significant time windows, respectively [−3, −2.5] S, [−0.5, 0] S and [0, 0.5] S.
