# Supplementary material for: Sensorimotor Oscillations in Human Infants during an Innate Rhythmic Movement
Source: Brain Sci. 2024 Apr 20;14(4):402. doi: 10.3390/brainsci14040402 (PMC11047852; doi:10.3390/brainsci14040402)
Supplement: Supplementary file 1 [file brainsci-14-00402-s001.zip › brainsci-2916586-supplementary.pdf]

Supplementary Materials: The following supporting information can be downloaded at: www.mdpi.com/xxx/s1.

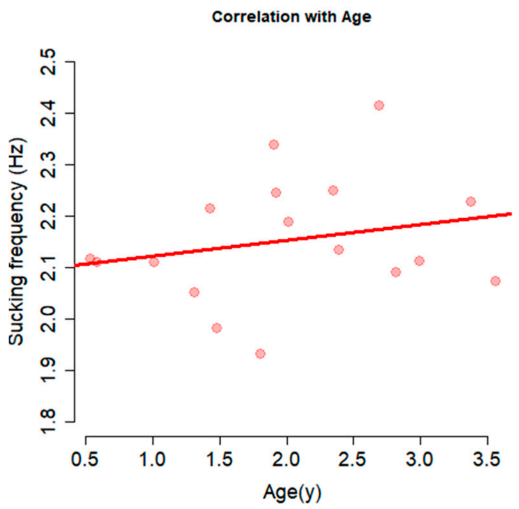

Figure S1. Correlation of sucking frequency with age. No significant correlation was found between sucking frequency (Hz) and the subjects age (y), R^2= 0.05, p=0.4. Points represent individual subjects, while continuous line represents the best linear fit.

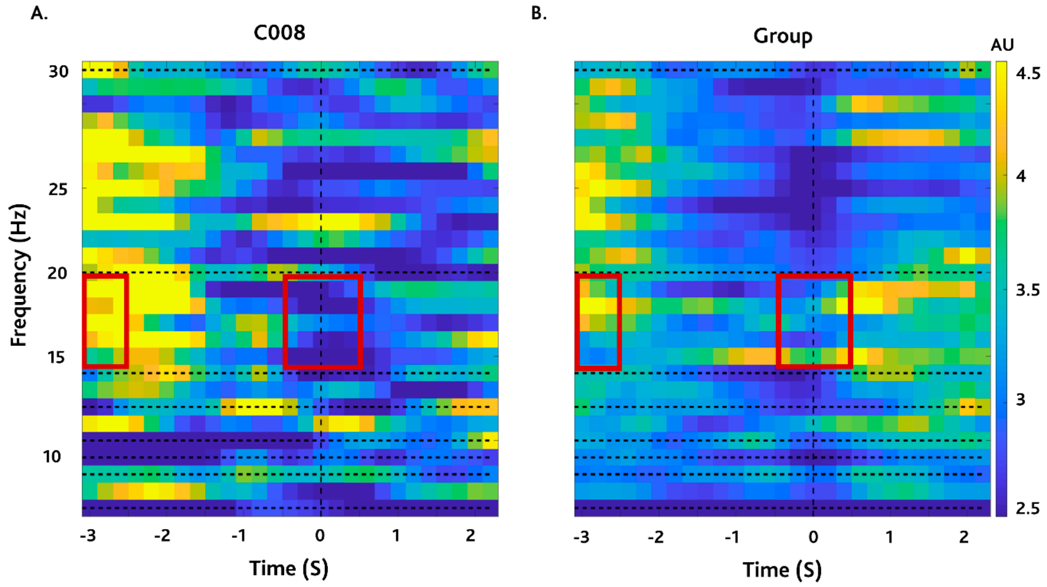

Figure S2. Time-frequency plot. x-axes represent time (S) and y-axes frequency (Hz). The red lines delimit the time-frequency bins resulted significant from the linear mixed model analyses. (**A**) Example of heat map obtained from typical subject (C008, age= 1.9). (**B**) Heat map that includes all subjects.

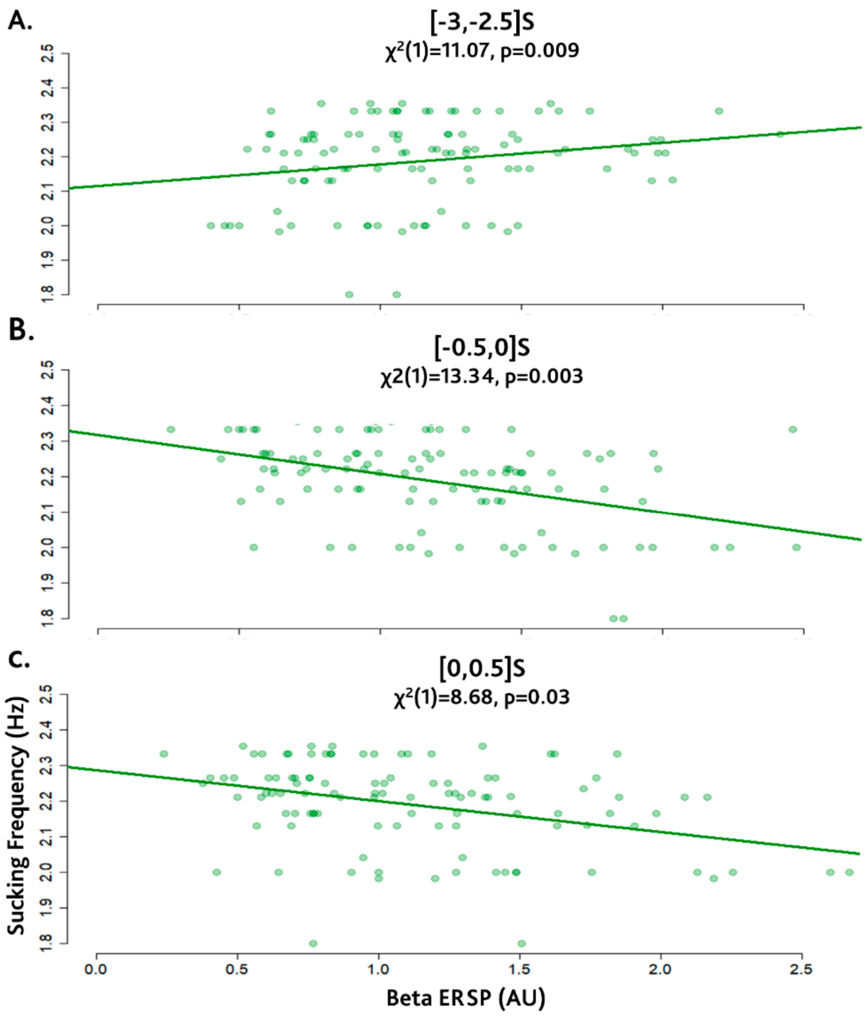

Figure S3. Correlations for each time window. Significant associations were found between relative beta ERSP (arbitrary units, AU) and sucking frequency (Hz). Each point represents a single trial, while continuous lines represent the best linear fit. (**A**), (**B**) and (**C**) show the associations in the significant time windows, respectively [-3,-2.5]S, [-0.5,0]S and [0,0.5]S.

Table S1. Individual subject data. Individual subject data at different ages showing the specific non-nutritive sucking (NNS) frequency and relative power of mu and beta activity during baseline and sucking period.

				**Baseline relative power (%)**			**NNS relative power (%)**		
**Code**	Fractional age	Biological Sex	SF (Hz)	μ1	μ2	β	μ1	μ2	β
**C001**	0.53	M	2.12	0.6862	0.4086	0.2108	0.4570	0.2852	0.1113
**C002**	0.58	F	2.11	0.4403	0.3643	0.0977	0.4484	0.2318	0.1280
**C003**	1.01	F	2.11	0.5777	0.2641	0.1913	0.5195	0.2135	0.0981
**C004**	1.31	F	2.05	1.3126	0.3757	0.1634	1.5741	0.7329	0.1301
**C005**	1.43	F	2.22	4.0068	0.4753	0.2819	3.4500	0.3963	0.2161
**C006**	1.48	F	1.98	1.9150	0.8491	0.3739	0.5678	0.2857	0.1175
**C007**	1.80	M	1.93	1.7881	0.3942	0.2710	0.5215	0.2660	0.1148
**C008**	1.90	F	2.34	3.2554	0.8381	0.3822	3.7801	0.8326	0.3742
**C009**	1.92	M	2.25	1.5316	0.5621	0.1690	1.9951	0.9746	0.2386
**C010**	2.01	M	2.19	0.7811	0.3063	0.1382	1.1779	0.3390	0.1442
**C011**	2.35	M	2.25	1.3074	0.5039	0.3428	0.7736	0.3587	0.2283
**C012**	2.39	M	2.13	2.3704	0.4849	0.3376	4.5930	0.8096	0.3907
**C013**	2.69	M	2.41	5.5940	0.4866	0.1913	4.9814	0.8331	0.3641
**C014**	2.82	M	2.09	1.7985	0.7566	0.3534	1.5006	0.3924	0.1743
**C015**	2.99	M	2.11	2.3689	0.9839	0.3029	2.8824	1.7203	0.3129
**C016**	3.38	M	2.23	1.7705	0.7795	0.3834	0.9044	0.4201	0.2771
**C017**	3.56	M	2.07	1.2630	0.7858	0.3636	1.2456	0.7292	0.3113
